# Preliminary evaluation of a new prototype interferon-gamma release assay for the detection of *Mycobacterium tuberculosis*–specific T-cell responses in patients with tuberculosis

**DOI:** 10.1007/s12223-025-01252-w

**Published:** 2025-03-03

**Authors:** Markéta Ibrahimová, Karolína Doležalová, Luboš Bača, Mariia Sukholytka, Evelin Grage-Griebenow, Dorinja Zapf, Sandra Saschenbrecker, Victor Herbst, Emilia Kopecká, Martina Koziar Vašáková

**Affiliations:** 1 Laboratory of Immunology, Thomayer University Hospital, Prague, Czech Republic; 2 Department of Pediatrics, First Faculty of Medicine, Charles University and Thomayer University Hospital, Prague, Czech Republic; 3 Department of Respiratory Medicine, First Faculty of Medicine, Charles University and Thomayer University Hospital, Prague, Czech Republic; 4 Institute for Experimental Immunology, affiliated to EUROIMMUN Medizinische Labordiagnostika AG, Lübeck, Germany

**Keywords:** IFN-γ, IGRA, Interferon-gamma release assay, *Mycobacterium tuberculosis*, Tuberculosis disease, Tuberculosis infection, T-cell response

## Abstract

Screening for tuberculosis infections (TBI) using the tuberculin skin test or interferon-gamma release assays (IGRA) is crucial in controlling the global TB burden. This study evaluates the performance of a new IGRA for the detection of T-cell responses against *Mycobacterium tuberculosis*. Blood samples from 34 adults with tuberculosis disease (TB) and from 30 children with TB, TBI or without TB were analyzed using the prototype Quan-T-Cell TB (EUROIMMUN). The pediatric samples were additionally measured using the established QuantiFERON-TB Gold Plus assay (Qiagen). Clinical performance and inter-assay concordance were analyzed. The prototype Quan-T-Cell TB yielded positivity rates of 88.2% and 100% in adults with TB and children with TBI, respectively, at a specificity of 93.8%. Comparison between the two IGRAs showed positive, negative and overall agreement rates of 100%, 93.8% and 96.3%, respectively, with a kappa score of 0.924 indicating almost perfect agreement. Our study shows promising results of the new prototype Quan-T-Cell TB, as reflected by high concordance with the final diagnosis in adults and children and performance comparable to that of the QuantiFERON IGRA. In individual cases, the data suggest that the prototype Quan-T-Cell TB may be even more consistent with TBI-related clinical findings. Unlike the QuantiFERON assay, the Quan-T-Cell TB has a predefined borderline range, which is advantageous as it may help to differentiate non-specific variation near the cut-off, and fewer sample tubes are required per analysis. The new Quan-T-Cell TB may therefore be a good alternative to the established QuantiFERON IGRA for TBI screening. Further assay optimization is underway, including evaluation studies based on larger patient and control cohorts.

## Introduction

Tuberculosis disease (TB), an airborne illness caused by *Mycobacterium tuberculosis* (MTB), is still a major public health problem, presenting one of the leading infectious causes of death worldwide (1.3 million fatalities in 2022) (World Health Organization (WHO) 2023). TB primarily affects the lungs (pulmonary TB), but approximately 20–30% of TB patients have extrapulmonary disease in either combination with the lungs or as a sole presentation (Gopalaswamy et al. 2021). MTB infection can result in a spectrum of responses and outcomes (Lin and Flynn 2018). The majority of those infected develop TB infection (TBI, LTBI = latent TBI in previous terminology), which is characterized by persistent immunoreactivity to MTB in the absence of clinical and radiologic TB manifestations (Behr et al. 2021). TBI can persist for weeks, months or years before developing into active contagious disease. Without treatment, approximately 4–6% of those who acquire TBI will develop TB, and the risk of progression is highest within 2 years following exposure (Lewinsohn et al. 2017).

The primary objective in the management of TB is the identification and treatment of persons with active TB, coupled with the screening of their contacts (Cole et al. 2020). Diagnosis of pulmonary TB is based on clinical examination and chest radiography. Sputum culture with microbiological confirmation remains the gold standard for diagnosing TB in adults. In addition, molecular methods (polymerase chain reaction [PCR]) allow for rapid identification of mycobacterial DNA and drug resistance testing (Lewinsohn et al. 2017; Lin and Desmond 2014).

Another priority of global TB control strategies is the timely identification and prophylactic therapy of symptom-free persons who are latently infected to prevent these cases from developing and transmitting active disease, as well as to reduce TB-related morbidity and mortality (Cohen et al. 2019; Cole et al. 2020; Getahun et al. 2015). Hence, TBI screening is important for different risk groups including contact persons (household contacts, healthcare workers, prisoners, etc.) (Pai et al. 2014; Redelman-Sidi and Sepkowitz 2013). For example, it is imperative to exclude TBI prior to the commencement of immunosuppressive therapy (e.g. tumor necrosis factor alpha [TNFα] inhibitors) in patients presenting with a multitude of immune-mediated inflammatory disorders, including inflammatory bowel diseases, rheumatological inflammations and multiple sclerosis. This is because the administration of anti-TNFα agents carries a fivefold increased risk of progressing from TBI to TB (Greveson et al. 2011). In the case of proven TBI, the introduction of a TB-preventive therapy is recommended (Santoro-Lopes et al. 2018; Selwyn et al. 1989).

The tuberculin skin test (TST) and T-cell interferon-γ release assays (IGRAs) represent the primary screening tools for TBI (Lewinsohn et al. 2017). As indirect immunological methods, these tests give evidence of contact with MTB, but they do not enable accurate distinction between latent or active disease, de novo or treated infection, recent (< 2 years) or past infection (Doležalová et al. 2024a; Getahun et al. 2015; Herrera et al. 2011).

The TST measures the immune reaction following intradermal injection of purified protein derivative (PPD) of MTB (Andersen et al. 2000). Due to cross-reactivity of PPD with bacille Calmette-Guérin (BCG) vaccine or non-tuberculous mycobacteria (NTM) antigens, the TST has limited specificity in vaccinees, and in populations with high prevalence of NTM infection and low prevalence of MTB infection (Farhat et al. 2006; Latorre et al. 2010; Mancuso et al. 2017; Nolt and Starke 2021). False-negative TST reactions may occur in immunocompromised patients, anergic persons, children (< 6 months), after very recent MTB infection, and recent live-virus measles or smallpox vaccination. Further limitations of the TST include inter-observer variability, the necessity of a revisit, and its potential to cause or increase T-cell and IGRA responses (“boosting”) that might be misinterpreted as a new infection (Centers for Disease Control and Prevention (CDC) 2020; Herrera et al. 2011; van Zyl-Smit et al. 2009).

In contrast to TST, IGRAs measure the secretion of IFN-γ by MTB-specific T effector cells. Two methodically different types of commercial IGRAs are available: the ELISPOT-based T-SPOT.TB (Oxford Immunotec/Revvity, Abingdon, UK) and the ELISA/LIAISON-based QuantiFERON-TB Gold Plus (QFT-Plus; Qiagen, Hilden, Germany) (Andersen et al. 2000). IFN-γ release is determined in response to stimulation with two highly immunogenic and specific MTB antigens, i.e. early secretory antigenic target 6 (ESAT-6) and culture filtrate protein 10 (CFP-10), either in peripheral blood mononuclear cells (T-SPOT.TB) or in whole blood (QFT-Plus) (Andersen et al. 2000; Fox et al. 2007). ESAT-6 and CFP-10 are absent in BCG and in most NTM strains, resulting in a higher specificity of IGRAs compared with the TST (Andersen et al. 2000; Mazurek et al. 2001; Pai et al. 2014). Further advantages of IGRAs relate to logistics (no revisit), rapid turnaround time, laboratory-based processing following standardized operational procedures, objective readout and no boost effect, as well as inclusion of positive and negative controls (European Centre for Disease Prevention and Control (ECDC) 2011; Greveson et al. 2011; Huang et al. 2016). On the other hand, IGRAs have limitations such as conduction within a given time frame after blood draw, higher resource demands (e.g. laboratory, personal, reagents), unknown prognostic value and limited interpretive criteria (European Centre for Disease Prevention and Control (ECDC) 2011; Herrera et al. 2011; Pai et al. 2014). However, not least by reason of high specificity, (inter)national guidelines have included IGRAs as confirmatory test in TST-positive cases or as TST substitute for diagnosing TBI (Mazurek et al. 2010). Moreover, even though IGRAs are not directly diagnostic for TB, clinicians may use them to obtain additional information as part of the diagnostic work-up in specific clinical situations (e.g. children with paucibacillary TB, patients with extrapulmonary TB, patients who test negative for acid-fast bacilli in sputum and/or negative for MTB on culture) (European Centre for Disease Prevention and Control (ECDC) 2011; Sester et al. 2011).

Here, we present the results of a pilot study that evaluated the performance of a new ELISA-based prototype IGRA for the detection of MTB-specific T-cell responses in whole-blood samples. In the absence of an adequate gold standard assay for the diagnosis of TBI, surrogate reference standards were used to assess accuracy in cohorts of confirmed TB cases (Hamada et al. 2021).

## Materials and methods

### Patients

**Fig. 1:**
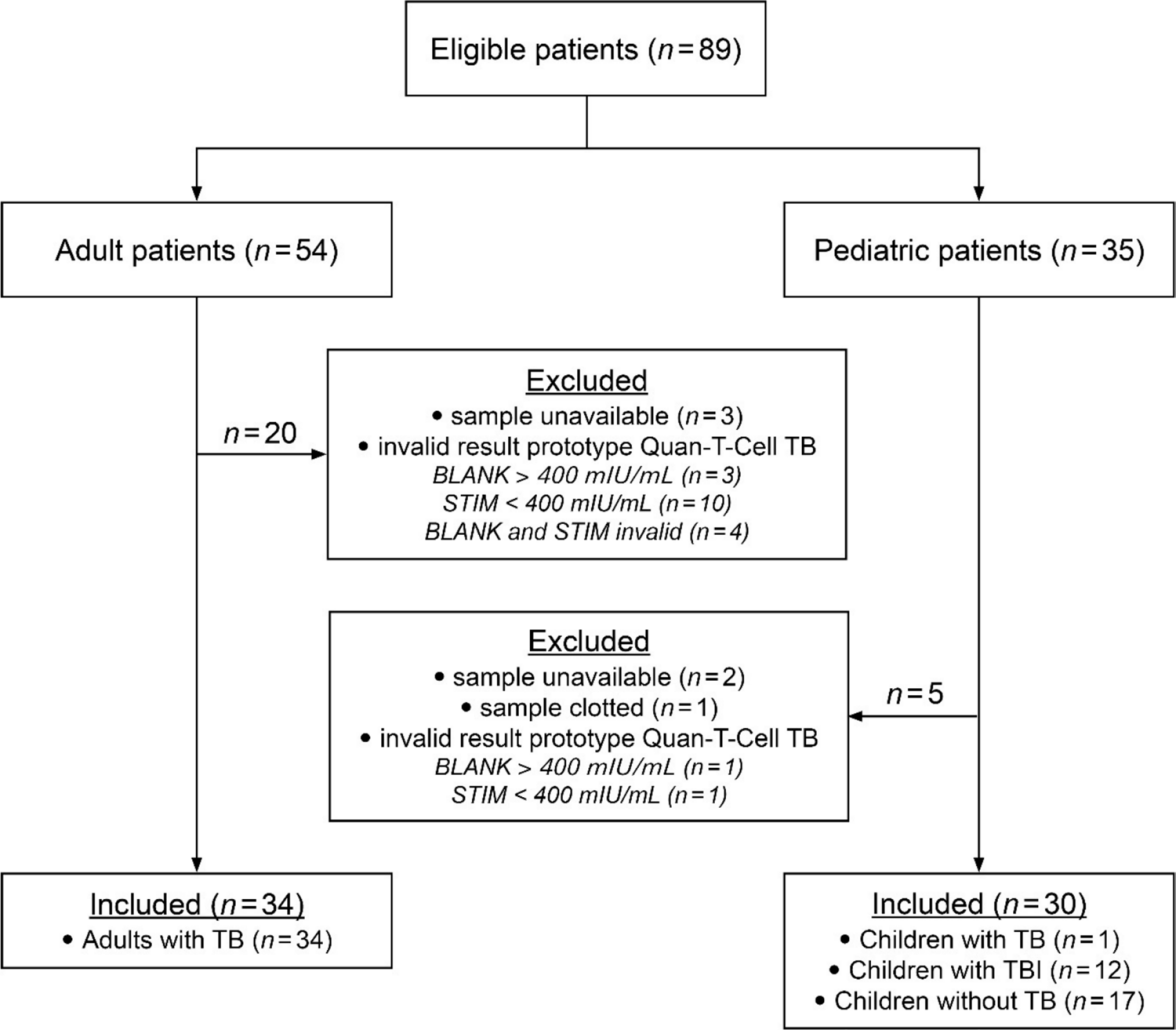
Flow chart summarizing the recruitment and diagnostic classification of study participants. *BLANK* negative control without stimulating components, *STIM* unspecific positive control coated with mitogen, *TB* tuberculosis disease, *TBI* tuberculosis infection

The study participants were recruited at the Department of Respiratory Medicine and the Department of Pediatrics at the Charles University and Thomayer University Hospital (Prague, Czech Republic): [i] adults diagnosed with TB and [ii] children in close contact with pulmonary TB (partly under treatment with isoniazide and rifampicin), children with symptoms typical for TB, or proven TB, children screened for TB before starting or continuing TNFα therapy (some on immunosuppressive therapy with, e.g., methotrexate or etanercept). The diagnosis of TB and TBI was made in accordance with established procedures and guidelines (Doležalová et al. 2024b; Vašáková 2013), including results from chest radiography, microbiological culture methods, microscopy, PCR, TST and IGRA (QFT-Plus), as appropriate. Whole-blood samples were collected from all study participants and subjected to parallel testing with the QFT-Plus and the EUROIMMUN prototype Quan-T-Cell TB IGRA. Of 89 subjects considered for the study, six were excluded due to unavailable or clotted samples and 19 due to invalid Quan-T-Cell TB results. The final study population comprised 34 adults, all of whom had been clinically diagnosed with TB, and 30 children, of whom one had TB, 12 had TBI and 17 developed neither TB nor TBI (Fig. 1). The characteristics of each patient group are summarized in Table 1.

**Table 1:** Demographics and clinical information of the study participants

Characteristics		Adults	Children		
		TB	TB	TBI	Without TB
Number	*n*	34	1	12	17
Sex	Female, *n* (%)	11 (32.4)	1 (100)	7 (58.3)	8 (47.1)
	Male, *n* (%)	23 (67.6)	―	5 (41.7)	9 (52.9)
Age	Mean ± SD, years	43.4 ± 11.3	0	7 ± 2.4	9.4 ± 4.1
	Median, years	42.5	0	7.5	10.0
	Range, years	23–66	―	3–10	2–15
BCG vaccination	Yes, *n* (%)	34 (100)	―	6 (50.0)	7 (41.2)
	No, *n* (%)	―	1 (100)	2 (16.7)	3 (17.6)
	Unknown	―	―	4 (33.3)	7 (41.2)
TB exposure	*n* (%)	34 (100)	1 (100)	12 (100)	10 (58.8)
	Unknown	―	―	―	7 (41.2)
TST	Positive, *n* (%)	―	1 (100)	4 (33.3)	1 (5.9)
	Negative, *n* (%)	―	―	―	11 (64.7)
	ND	34 (100)	―	8 (66.7)	5 (29.4)
Chest X-ray	Positive, *n* (%)	34 (100)	1 (100)	―	―
	Negative, *n* (%)	―	―	12(100)	17 (100)
Culture^a^	Positive, *n* (%)	30 (88.2)	―	―	―
	Negative, *n* (%)	4 (11.8)	―	―	―
	ND	―	1 (100)	12 (100)	17 (100)
Microscopy^b^	Positive, *n* (%)	20 (58.8)	―	―	―
	Negative, *n* (%)	14 (41.2)	―	―	―
	ND	―	1 (100)	12 (100)	17 (100)
PCR^c^	Positive, *n* (%)	30 (88.2)	―	―	―
	Negative, *n* (%)	4 (11.8)	―	―	―
	ND	―	1 (100)	12 (100)	17 (100)
IGRA^d^	Positive, *n* (%)	―	―	11 (91.7)	―
	Negative, *n* (%)	―	―	1 (8.3)	17 (100)
	ND	34 (100)	1 (100)	―	―
TB/TBI therapy	Yes, *n* (%)	34 (100)	1 (100)	12 (100)	―
	No, *n* (%)	―	―	―	17 (100)

*BCG* bacille Calmette-Guérin, *IGRA* interferon-gamma release (IFN-γ) assay, *ND* not determined, *PCR* polymerase chain reaction, *SD* standard deviation, *TB* tuberculosis disease, *TBI* tuberculosis infection, *TST* tuberculin skin test

^a^Sputum samples were tested using classical culture and Bact/BACTEC liquid culture (BD BACTEC™ MGIT™ 960 (Becton-Dickinson, Fanklin Lakes, NJ, USA). ^b^Sputum smear was tested after Ziehl-Neelsen staining. ^c^Sputum samples were tested using the Xpert MTB/RIF assay performed on the GeneXpert system (Cepheid Inc., Sunnyvale, CA, USA). ^d^Whole-blood samples were tested for IFN-γ release using the QuantiFERON-TB Gold Plus (QFT-Plus) assay (Qiagen, Hilden, Germany)

### Prototype Quan-T-Cell TB

Whole-blood specimens were collected by venipuncture into lithium heparin tubes and stored no longer than 16 h at room temperature, followed by transfer into the Quan-T-Cell TB stimulation tubes (EUROIMMUN Medizinische Labordiagnostika AG, Lübeck, Germany), as recommended by the manufacturer. Per analysis, 1.5 mL blood (three tubes of 0.5 mL each) were required.

The Quan-T-Cell TB prototype assay (EUROIMMUN) contains stimulation tube sets, each consisting of three tubes per patient: (I) BLANK (negative control without stimulating components), (II) STIM (unspecific positive control coated with mitogen) and (III) TB-specific stimulation (coated with stimulatory antigens that are based on mycobacterial proteins ESAT-6 and CFP-10). Before use, all tubes were brought to room temperature for 30 min. Per patient, 0.5 mL of heparinized whole blood was pipetted into each of the three tubes and mixed by inverting, followed by incubation at 37 °C for 20–24 h to allow T-cell stimulation. After centrifugation at 12,000 × g for 10 min, the supernatant plasma was stored at 4 °C (≤ 28 days) or at −20 °C until IFN-γ measurement. The concentration of released IFN-γ was determined using the Quan-T-Cell ELISA (EUROIMMUN), which is based on microplates coated with monoclonal anti-IFN-γ antibodies. The plates were processed on the DSX Automated ELISA System (Dynex Technologies, Chantilly, VA, USA) according to the manufacturer’s instructions. Briefly, plasma samples diluted 1:5 in sample buffer were added to the wells and allowed to react at room temperature for 120 min. After a wash cycle, a solution containing biotin-labelled anti-IFN-γ antibodies was added for 30 min, followed by washing, incubation with peroxidase-labelled streptavidin for 30 min and another wash cycle. A chromogen/substrate solution (tetramethylbenzidine/H_2_O_2_) was added to induce a color reaction for 20 min. The enzymatic reaction was stopped with 0.5 mol/L sulphuric acid. Absorbance was measured photometrically at 450 nm (reference 620 nm). Calibrators, negative and positive controls were included in each test run. Using a 6-point calibration curve, IFN-γ levels were quantified in mIU/mL. To exclude processing errors, the BLANK and STIM results of each sample were checked for validity criteria: samples with invalid data (BLANK > 400 mIU/mL, STIM minus BLANK < 400 mIU/mL) were excluded from evaluation, as recently described for the Quan-T-Cell ELISA (Infantino et al. 2022). For MTB-specific stimulation, IFN-γ levels (minus BLANK) > 200 mIU/mL were considered positive, between 100 and 200 mIU/mL borderline and values < 100 mIU/mL negative (Infantino et al. 2022).

### QuantiFERON-TB Gold Plus

Whole blood was collected directly into QFT-Plus blood collection tubes (Qiagen, Hilden, Germany), as recommended by the manufacturer (Qiagen 2024a). Per analysis, 4.0 mL blood (four tubes of 1.0 mL each) was required.

The QFT-Plus (Qiagen, Hilden, Germany) was used according to the manufacturer’s instructions (Qiagen 2024b). As recommended by the manufacturer, IFN-γ values ≥ 0.35 IU/mL (after nil subtraction) were considered positive. QFT-Plus testing was limited to samples from children with TBI and without TB, and only qualitative QFT-Plus results were reported.

### Statistics

Confidence intervals [95% CI] were calculated using the method of Clopper and Pearson. Inter-assay concordance was assessed using percentage of agreement between qualitative results. To correct the agreement for the probability of random coincidence, Cohen’s kappa coefficient was calculated. According to Landis and Koch, kappa values from 0 to 0.20, 0.21 to 0.40, 0.41 to 0.60, 0.61 to 0.80 and 0.81 to 1.00 indicate slight, fair, moderate, substantial and near-perfect agreement, respectively (Landis and Koch 1977). For the calculation of accuracy and concordance, samples with borderline Quan-T-Cell TB results (accounting for 2/64 [3.1%] cases) were excluded. Differences between the diagnostic groups were analyzed using Fisher’s exact test for categorical data and the Mann-Whitney *U* test for continuous variables (non-normal distribution in all panels according to the Shapiro-Wilk test). *p* < 0.05 was considered to indicate a statistically significant difference. Statistical analyses were conducted using SigmaPlot 13.0 (SSI, San Jose, CA) and GraphPad QuickCalcs (GraphPad Software, Inc., San Diego, CA).

## Results

### Prototype Quan-T-Cell TB: quantitative results

**Fig. 2:**
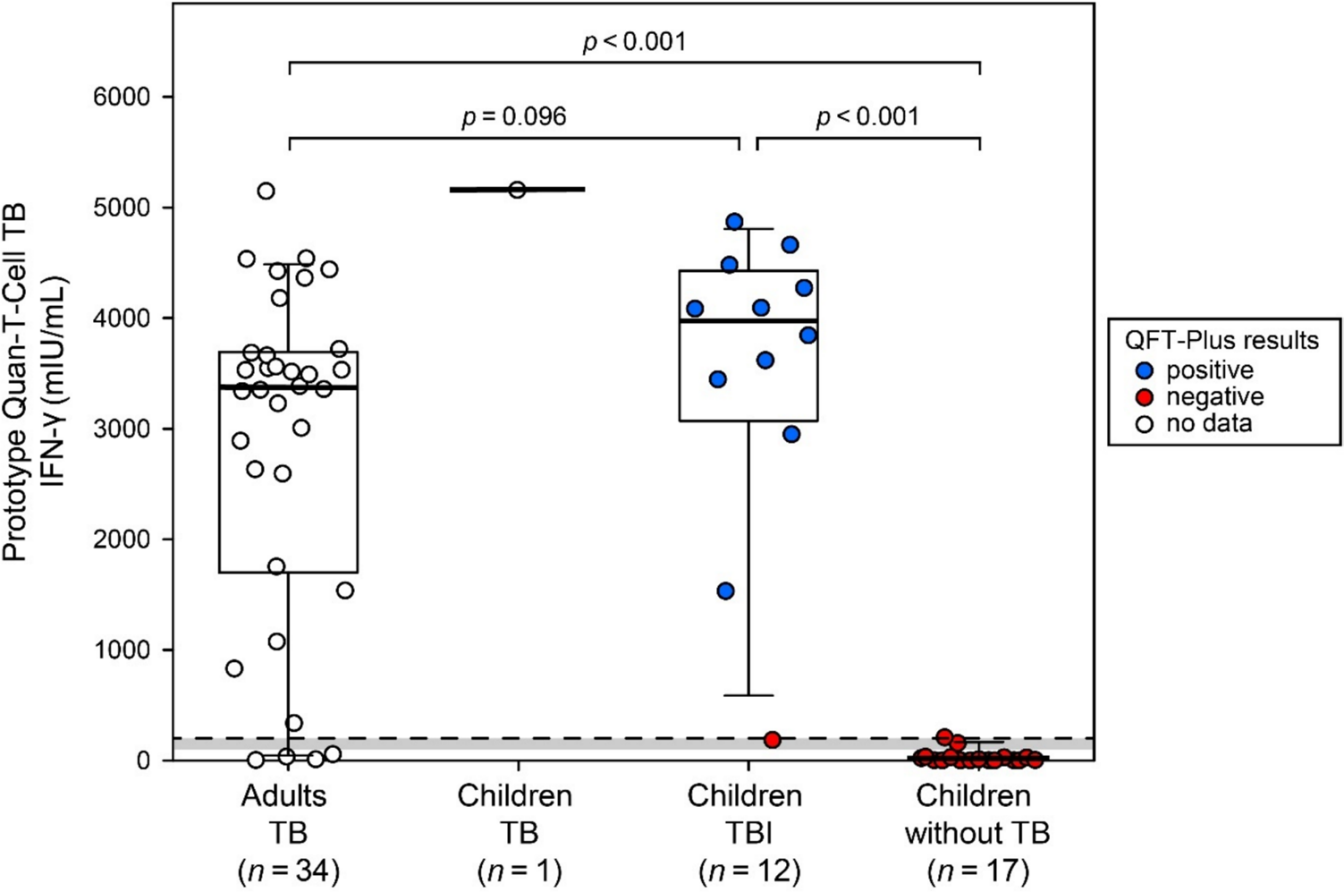
Scattered boxplots displaying interferon-gamma (IFN-γ) release in adult patients and children. IFN-γ levels were measured using the prototype Quan-T-Cell TB assay. Boxes indicate interquartile ranges (outer bounds) and medians (midlines). Whiskers present the 90th and 10th percentiles. The dashed line represents the cut-off for positivity (> 200 mIU/mL). The borderline range (100–200 mIU/mL) is shaded in grey. Mean IFN-γ levels were compared between groups (*n* > 1) using the Mann-Whitney *U* test. Data points are colored based on the qualitative results obtained with the QuantiFERON-TB Gold Plus (QFT-Plus) assay, as indicated in the legend. *TB* tuberculosis disease, *TBI* tuberculosis infection

INF-γ release after MTB-specific antigen stimulation was measured quantitatively using the prototype Quan-T-Cell TB in all four patient groups. Results are summarized in Table 2. The mean levels of released INF-γ showed no significant difference between adults with TB and children with TBI (*p* = 0.096). However, INF-γ release was significantly higher in these two groups than in children without TB (*p* < 0.001, Fig. 2).

**Table 2:** Quantitative determination of INF-γ release determined using the prototype Quan-T-Cell TB in the different groups of patients

Group of patients	*n*	Prototype Quan-T-Cell TB (EUROIMMUN), in mIU/mL					
		Mean	SD	Min	Max	Median	IQR
Adults, TB	34	2861.2	1504.1	3.1	5151.6	3371.0	1698.4–3694.4
Children, TB	1	5157.1	―	5157.1	5157.1	5157.1	5157.1–5157.1
Children, TBI	12	3502.4	1374.9	183.1	4869.9	3964.2	3073.2–4427.9
Children, without TB	17	29.8	59.2	0.5	208.2	3.3	0.5–27.5

*IQR* interquartile range, *Max* maximum, *Min* minimum, *SD* standard deviation, *TB* tuberculosis disease, *TBI* tuberculosis infection

### Prototype Quan-T-Cell TB and QFT-Plus: qualitative results

In the group of adults with TB, the prototype Quan-T-Cell TB was INF-γ-positive in 30/34 cases, indicating a diagnostic sensitivity of 88.2%. Excluding one TBI case with a borderline result, all children diagnosed with TB or TBI were tested INF-γ-positive (100%, 12/12). Referring to 17 children without TB, the prototype Quan-T-Cell TB results were borderline or very low positive in one case each, whereas 15 children were negative, indicating a specificity of 93.8% (15/16, excluding the borderline case, Table 3). Comparison between groups with more than one case revealed that the rate of patients with positive INF-γ results was not significantly different between adults with TB and children with TBI (*p* = 0.558), while significantly more patients in these two groups had positive results compared with children without TB (*p* < 0.001).

**Table 3:** Performance characteristics of INF-γ release assays

Panel	*n*	Prototype Quan-T-Cell TB (EUROIMMUN)					QuantiFERON-TB Gold Plus (Qiagen)			
		Pos	Bdl	Neg	Positivity rate [95% CI]	Specificity [95% CI]	Pos	Neg	Positivity rate [95% CI]	Specificity [95% CI]
Adults, TB	34	30	0	4	88.2% [72.6%, 96.7%]	―	ND	ND	―	―
Children, TB	1	1	0	0	100% [2.5%, 100%]	―	ND	ND	―	―
Children, TBI	12	11	1^a^	0	100%^d ^[71.5%, 100%]	―	11	1^a^	91.7% [61.5%, 99.8%]	―
Children, without TB	17	1^b^	1^c^	15	―	93.8%^d ^[69.8%, 99.8%]	0	17	―	100% [80.5%, 100%]

*BCG* bacillus Calmette-Guérin, *Bdl* borderline, *CI* confidence interval, *ND* not determined, *Neg* negative, *Pos* positive, *QFT-Plus* QuantiFERON-TB Gold Plus assay, *TB* tuberculosis disease, *TBI* tuberculosis infection, *TST* tuberculin skin test

^a^7-year-old boy diagnosed with TBI: chest X-ray negative, TST positive, QFT-Plus negative, without BCG vaccination, contact with a patient with TB, undergoing TBI treatment for 3 months. ^b^6-year-old boy diagnosed without TB: chest X-ray showing hilar lymphadenopathy, TST negative, QFT-Plus negative, without BCG vaccination, undergoing treatment with cough-suppressant. ^c^14-year-old boy diagnosed without TB: chest X-ray negative, TST not determined, QFT-Plus negative, BCG vaccination 14 years ago, contact with a patient with TB. ^d^Borderline results were excluded from accuracy analysis

Using the QFT-Plus assay, 91.7% (11/12) of samples from children with TBI showed a positive response, while all 17 samples from children without TB were negative (100% specificity) (Table 3, Fig. 2).

### Comparison Quan-T-Cell TB versus QFT-Plus

As samples from patients with TB were not analyzed using the QFT-Plus, the analysis of inter-assay agreement was restricted to 29 samples from children with TBI and children without TB. All 11 samples with an INF-γ-positive result by QFT-Plus were also positive by the prototype Quan-T-Cell TB, corresponding to a positive agreement rate of 100% (11/11). Out of 18 samples classified as negative by QFT-Plus, 15 tested negative, one positive and two borderline using the Quan-T-Cell TB, resulting in a negative agreement rate of 93.8% (15/16) excluding borderline results. The overall agreement amounted to 96.3% based on the concordance of positive and negative results in 26/27 cases, with a kappa score of 0.924 indicating almost perfect agreement between the two IGRAs (Table 4).

**Table 4:** Agreement of qualitative results between the prototype Quan-T-Cell TB and QuantiFERON-TB Gold Plus assay in blood samples from children with TBI (*n* = 12) and children without TB (*n* = 17)

Prototype Quan-T-Cell TB (EUROIMMUN)	QuantiFERON-TB Gold Plus (Qiagen)		
	Positive	Negative	Total
Positive	11	1^a^	12
Borderline	0	2^b,c^	2
Negative	0	15	15
Total	11	18	29
Positive agreement [95% CI]	100% [71.5%, 100%]		
Negative agreement [95% CI]^d^	93.8% [69.8%, 99.8%]		
Overall agreement [95% CI]^d^	96.3% [81.0%, 99.9%]		
Cohen’s kappa [95% CI]^d^	0.924 [0.779, 1.000]		

*BCG* bacillus Calmette-Guérin, *CI* confidence interval, *QFT-Plus* QuantiFERON-TB Gold Plus assay, *TB* tuberculosis disease, *TBI* tuberculosis infection, *TST* tuberculin skin test

^a^7-year-old boy diagnosed with TBI: chest X-ray negative, TST positive, QFT-Plus negative, without BCG vaccination, contact with a patient with TB, undergoing TBI treatment for 3 months. ^b^6-year-old boy diagnosed without TB: chest X-ray showing hilar lymphadenopathy, TST negative, QFT-Plus negative, without BCG vaccination, undergoing treatment with cough-suppressant. ^c^14-year-old boy diagnosed without TB: chest X-ray negative, TST not determined, QFT-Plus negative, BCG vaccination 14 years ago, contact with a patient with TB. ^d^Borderline results were excluded from accuracy analysis

## Discussion

This study is the first assessment of the performance characteristics of a new prototype TB IGRA, which is under development as an IVDR-approved test. The prototype Quan-T-Cell TB was compared to the established fourth-generation QFT-Plus assay, for which previous studies reported sensitivities of 85.0–95.8% for adults with TB (Shafeque et al. 2020), 82.1–98.3% for adults with TBI (Petruccioli et al. 2017; Theel et al. 2018), 54.5–87.3% for children with TB (Buonsenso et al. 2023) and 68.2–100% for children with TBI (Buonsenso et al. 2020; Soler-Garcia et al. 2022), at specificities ranging between 87.0% and 100% (Buonsenso et al. 2020; Shafeque et al. 2020). In line with this, a positivity rate of 91.7% (specificity 100%) was obtained for children with TBI using QFT-Plus in the current study. The prototype Quan-T-Cell TB revealed similar performance with positivity rates of 88.2% for adults with TB and 100% for children with TBI, at 93.8% specificity. Qualitative results obtained for pediatric samples were in near-perfect inter-assay agreement with the QFT-Plus (96.3%, kappa = 0.924). There were only three cases showing discordant results between the two IGRAs:

- Patient #1: A 7-year-old Romanian boy with a history of TB exposure was diagnosed with TBI (chest X-ray negative, TST positive) and treated with isoniazid/rifampicin for 3 months. The negative QFT-Plus result was inconsistent not only with the positive TST reaction, but also with the borderline result obtained with the Quan-T-Cell TB (183.1 mIU/mL).
- Patient #2: A 6-year-old Caucasian boy with chest X-ray showing hilar lymphadenopathy was diagnosed without TB. TST and QFT-Plus were both negative. The result of the prototype Quan-T-Cell TB was slightly positive (208.2 mIU/mL) and may reflect a higher sensitivity of this assay. Considering that hilar lymphadenopathy is observed in about 60% of children with primary TB (Kim et al. 2006), the low positive INF-γ levels measured with the Quan-T-Cell TB could also indicate a conversion caused by the onset of clinical disease.
- Patient #3: A 14-year-old Caucasian boy, BCG-vaccinated after birth, was tested for MTB infection after exposure to TB disease. Based on negative chest X-ray and negative QFT-Plus (TST not performed), he was diagnosed without TB. The prototype Quan-T-Cell TB result was borderline (155.5 mIU/mL).

In these three cases, low IFN-γ levels were detected using the prototype Quan-T-Cell TB (two cases with borderline results, one case with a weak positive result), while the QFT-Plus showed negative reactivity. Discrepancies between qualitative results may result from technical differences between the two IGRAs, such as the choice of stimulating antigens or the choice of the cut-off value. Like the QFT-Plus TB1 tubes, the Quan-T-Cell TB tubes contain ESAT-6- and CFP-10-derived antigens. The QFT-Plus assay includes a second antigen tube (TB2) containing processed ESAT-6/CFP-10 peptides that are optimized to elicit a CD8+ T-cell response in addition to the CD4+ response, with the intention to increase sensitivity based on higher CD8+ T-cell responses in cases of recent MTB exposure, TB and individuals with low CD4+ T-cell counts (TB/HIV-coinfection, children aged < 5 years) (Shafeque et al. 2020). However, studies elaborating these potential benefits of the QFT-Plus (compared to the QFT-GIT, a former version of the QFT-Plus) have not revealed any significant improvement in performance (Shafeque et al. 2020; Venkatappa et al. 2019; Yi et al. 2016). If the CD8+ T-cell responses can be neglected in the comparison between the prototype Quan-T-Cell TB and QFT-Plus, the choice of the predefined assay cut-offs for positive/negative classification is a more relevant explanation for the observed inter-assay discrepancies, just as the inclusion of a borderline range.

However, taking into consideration the above-mentioned clinical information (TST positivity, lymphadenopathy or TB contact) on the three patients with discordant IGRA readings, the borderline Quan-T-Cell TB results might also indicate higher sensitivity compared to the QFT-Plus which was negative in all these cases. Nevertheless, IGRAs with a grey zone for borderline results, like the prototype Quan-T-Cell TB and the T-SPOT.TB, must be interpreted cautiously within the clinical context and should entail follow-up testing. In contrast, the QFT assays have no manufacturer-predefined grey zone, although a borderline range or uncertainty zone covering results close to the cut-off (0.35 IU/mL) has been suggested repeatedly for low-risk individuals to allow a more definitive detection of MTB infection and to reduce the misinterpretation of cut-off-near minor variation in serial testing as true conversion (Herrera et al. 2011; Nemes et al. 2017). Several authors applied individual borderline ranges for QFT testing, showing that results within this range were partly explained by assay variability rather than true immunological responses (Metcalfe et al. 2013; Nemes et al. 2017; Schablon et al. 2014; Tagmouti et al. 2014). For example, Wikell et al. reported that 38% of samples with initial QFT-Plus-borderline results were clearly negative in follow-up testing within 4 weeks, indicating the relevance of a borderline range and repeated testing to avoid false-positive or false-negative results near the recommended cut-off (Wikell et al. 2021).

A drawback of IGRAs is the occurrence of indeterminate or invalid results, which cannot be interpreted, thus complicating clinical management and increasing the costs for additional testing. Invalid results may be obtained if certain conditions trigger the spontaneous IFN-γ release by T-cells in the absence of the mitogen (high value in negative control), or if the mitogen does not sufficiently stimulate the T-cells (low value in positive control), e.g. due to poor sample preparation, technical errors or qualitative defects in the lymphocyte response (Greveson et al. 2011; Lewinsohn et al. 2017). The reported overall rates of indeterminate QFT results amount to about 7% (range, 0–15%) in adult patients with TB (Pai and Menzies 2007; Santos et al. 2020), 4% in TBI screening (Zhou et al. 2023) and 4% in children (Meier et al. 2019). Significantly increased indeterminate rates occurred, e.g., in immunocompromised individuals, patients in intensive care units or subjects of old age (Ferrara et al. 2005; Huang et al. 2016; Santos et al. 2020; Zhou et al. 2023). In the present study, the prototype Quan-T-Cell TB showed invalid results at a rate of 31.5% in adults with TB and 5.7% in children (one case each with TBI and TB). It is unclear if the high rate observed for adults with TB also holds true for TBI screening, which is the major application of IGRAs. For the final assay to be used in clinical settings, a significantly lower invalidity rate is a mandatory requirement. It is therefore necessary to conduct further studies in order to identify the factors associated with invalid Quan-T-Cell TB results and to make the necessary adjustments to the assay’s invalidity thresholds. In this regard, it is necessary to determine whether different validity thresholds and positivity cut-offs should be applied to pediatric and adult samples.

As development and optimization of the Quan-T-Cell TB is currently in progress, additional elaborate evaluation studies will be needed. These should be based on larger, age- and sex-matched patient and control cohorts from different TB epidemiological settings, including adults with TBI and healthy donors, as well as serial testing (Hamada et al. 2021). In addition, comparisons should be made with other methods to support TBI diagnosis, like T-SPOT.TB and TST. As a result, the operating threshold values for validity, positivity and grey zone will be further validated and optimally adjusted in specific clinical contexts before approval of the final assay.

The prototype Quan-T-Cell TB can be particularly helpful in pediatric applications as it requires only 1.5 mL blood per analysis. In contrast, a minimum blood volume of 4 mL has to be obtained for the QFT-Plus assay, which is not always easy to collect in young children. Moreover, the requirement of only three tubes in the Quan-T-Cell TB compared to four tubes in the QFT-Plus not only minimizes the required sample volume but also increases the cost-effectiveness of laboratory testing.

This pilot study has several limitations. Firstly, this is a single-center study with a very low number of recruited participants. For this reason, the study lacks the statistical power needed to draw generalizable conclusions. Secondly, measurements were restricted to adult patients with TB and children with predominantly TBI or without TB, whereas adults with TBI and without TB were missing and children with TB were underrepresented. Disease control panels with relevance for differential diagnostics and healthy controls were not included either, limiting the validity of the diagnostic accuracy assessment. Finally, the prototype Quan-T-Cell TB was neither compared to the TST (in a non-BCG-vaccinated population) nor to the TB-SPOT.TB.

Recently, a second study analyzing the performance of the Quan-T-Cell TB has been conducted in Spain by Latorre et al. (2024), using samples from 23 adults with active TB (age, 41.6 ± 10.2 years), 29 adults undergoing TBI screening (48.9 ± 10.5 years) and 23 healthy blood donors (38.2 ± 13.5 years). Here, the positivity rates obtained with the Quan-T-Cell TB / QFT-Plus were 95.2% / 69.6% (TB) and 51.7% / 48.3% (screening TBI), at specificities of 100% (healthy controls). The assay agreement rate (90.3%) and kappa value (0.805) indicated almost perfect agreement. These results complement and support the findings of our study. It should be noted, however, that they also represent preliminary data obtained with the prototype assay in the development stage, and that their significance is limited due to the small number of patients.

## Conclusions

The preliminary results of this study suggest a promising performance of the prototype Quan-T-Cell TB as a test intended for TBI screening. Unlike the established QFT-Plus, the Quan-T-Cell TB has a predefined borderline range, which is advantageous as it may help to differentiate non-specific variation near the cut-off. Moreover, compared to the QFT-Plus, reduction to one MTB-specific stimulation tube and a lower volume per tube minimizes the required sample volume, and maximizes the cost-effectiveness of laboratory testing. Further optimization of the Quan-T-Cell TB is underway, requiring technical adjustments and additional evaluation studies. Importantly, careful storage and handling of blood samples as well as strict adherence to the incubation process specification are critical when performing IGRAs. The results should always be interpreted in the context of the patient’s clinical picture and contact history, and in conjunction with the data obtained from other laboratory diagnostic procedures. In cases of inconclusive or inconsistent results, other tests should be carried out in addition.

## Data Availability

The data presented in this study are available upon reasonable request from the corresponding author.

## Abbreviations

*BCG*: Bacille Calmette-Guérin
*CD*: Cluster of differentiation
*CFP-10*: Culture filtrate protein 10
*ELISA*: Enzyme-linked immunosorbent assay
*ESAT-6*: Early secretory antigenic target 6
*HIV*: Human immunodeficiency virus
*IGRA*: Interferon-gamma release assay
*IFN-γ*: Interferon-gamma
*IVDR*: In Vitro Diagnostic Medical Device Regulation
*MTB*: *Mycobacterium tuberculosis*
*NTM*: Non-tuberculous mycobacteria
*PCR*: Polymerase chain reaction
*PPD*: Purified protein derivative
*QFT*: QuantiFERON-TB
*QFT-GIT*: QuantiFERON-TB Gold In-Tube
*QFT-PLUS*: QuantiFERON-TB Gold Plus
*TB*: Tuberculosis disease
*TB-1*: Tube 1
*TB-2*: Tube 2
*TBI*: Tuberculosis infection
*TNFα*: Tumor necrosis factor alpha
*TST*: Tuberculin skin test
